# Endoscopic pancreatic duct stenting combined with 3D laparoscopic pancreatic tumor resection: Three case reports

**DOI:** 10.1097/MD.0000000000033733

**Published:** 2023-05-12

**Authors:** Bo Wu, Yang Bai, Shi’an Yu, Xuemin Li, Xiaokang Wu

**Affiliations:** a Department of Hepatobiliary and Pancreatic Surgery, Affiliated Jinhua Hospital, Zhejiang University School of Medicine, Jinhua, Zhejiang, PR China; b Department of Surgery, The Second Affiliated Hospital, Zhejiang University School of Medicine, Hangzhou, Zhejiang, PR China.

**Keywords:** endoscopic surgery, laparoscopic surgery, pancreatic duct stent, pancreatic tumor, 3D

## Abstract

This study explored the safety and feasibility of 3D laparoscopy and intraoperative ultrasound techniques, which made minimally invasive pancreatic surgery more precise and detailed.

**Patient concern:**

Case 1 was a 51-year-old man with a primary complaint of pancreatic tumor. Case 2 was a 60-year-old woman with complaints of tinnitus for 1 week. Case 3 was a 21-year-old woman with complaints of epigastric pain and abdominal distension for 1 day.

**Diagnosis:**

Case 1 and Case 2 were diagnosed with pancreatic neuroendocrine tumors, and Case 3 was diagnosed with an infected solid pseudopapillary tumor of the pancreas.

**Interventions:**

All 3 patients underwent laparoscopic pancreatic surgery in our hospital.

**Outcomes:**

All cases received the same perioperative management and no localized stenosis or dilatation of the pancreatic duct was found during follow-up.

**Lessons:**

With the development of minimally invasive surgery and the application of 3D laparoscopy and intraoperative ultrasound technology, pancreatic tumors that are tightly adhered to the main pancreatic duct can successfully be removed using 3D laparoscopic operation.

## 1. Introduction

Pancreatic tumor resection is the first choice for benign and borderline pancreatic tumors, which preserves the function of the pancreas and ensures long-term quality of life.^[1]^ However, the main pancreatic duct is easily damaged during denucleation.^[2]^ With the development of minimally invasive surgery and the application of 3D laparoscopy and intraoperative ultrasound technology, 3 patients with pancreatic tumors in close adhesion to the main pancreatic duct were successfully treated with 3D laparoscopic pancreatic tumor resection.

## 2. Case report

### 2.1. Case 1

**Figure 1.**
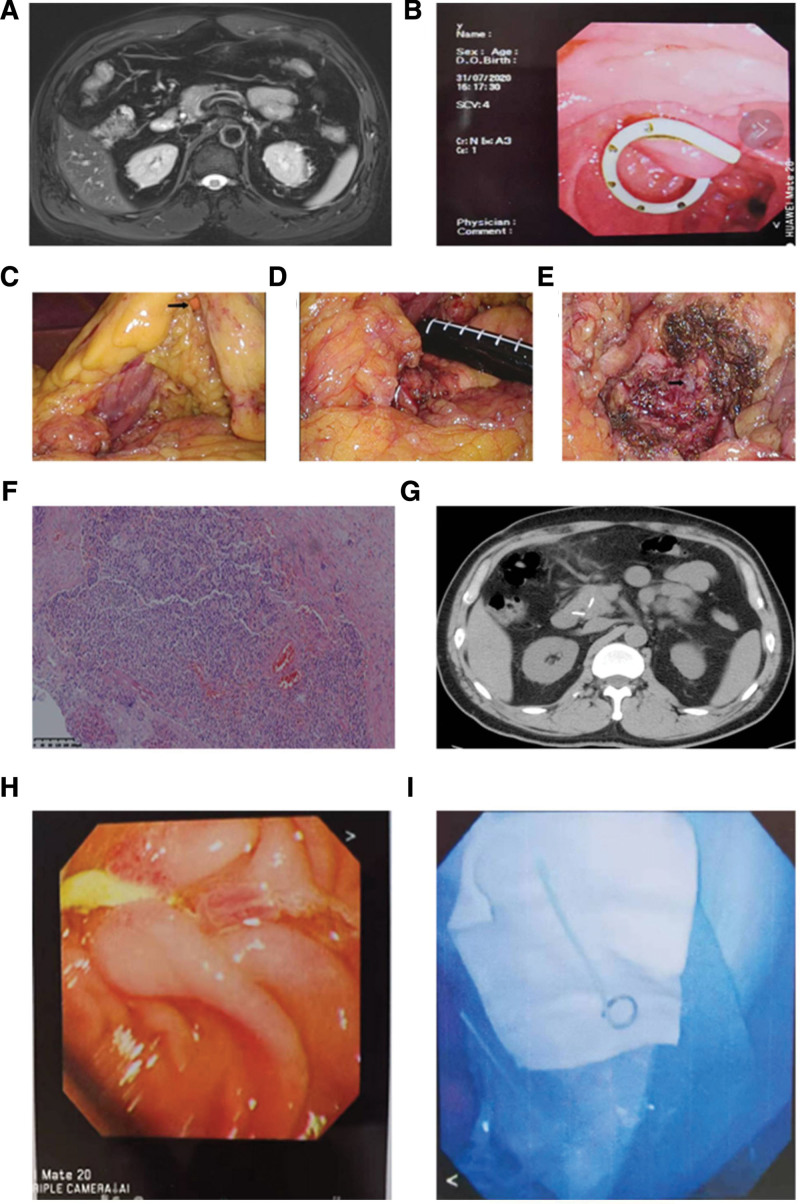
Imaging of the first patient. (A) Preoperative abdominal enhanced MRI found a pancreatic neck tumor with a slightly high signal focus on T2 and with clear boundaries close to the high signal pancreatic duct. (B) Pancreatic duct stent was placed. (C) The stomach was suspended during the surgery. (D) The tumor and pancreatic duct were located by intraoperative ultrasound. (E) Pancreatic incision after tumor enucleation. (F) Postoperative H&E pathological results. (G) The results of plain abdominal CT scan 1 month after surgery showed that the position of the pancreatic duct stent was good. (H) One month after the surgery, the pancreatic duct stent was removed. CT = computed tomography, H&E = hematoxylin and eosin, MRI = magnetic resonance imaging.

Case 1 was a 51-year-old man who was admitted to the hospital with a primary complaint of pancreatic tumor. Physical examination and hematologic findings were normal. Magnetic resonance cholangiopancreatography suggested a possible neuroendocrine tumor in the neck of the pancreas, measuring approximately 1.5 cm in length and <2 mm from the main pancreatic duct. Enhanced abdominal computed tomography (CT) showed an isointense nodule in the pancreatic neck, and magnetic resonance imaging (MRI) indicated the presence of a neuroendocrine tumor (Fig. 1A). The man was diagnosed with a nonfunctional pancreatic neuroendocrine tumor. As the patient wanted to surgically remove the tumor and maximize pancreatic function, endoscopic pancreatic duct stenting combined with 3D laparoscopic pancreatic tumor resection was ultimately chosen after being informed of the condition and surgical risks.

One day before the surgery, the pancreatic duct stent was placed using endoscopic retrograde cholangiopancreatography (ERCP). Under general anesthesia, the endoscope was placed into the descending duodenum. Then, a guide wire (Micro-Tech [Nanjing] Co., Ltd, Nanjing, China) was used to insert a catheter into the bile duct through the opening of the duodenal papilla. After incising the duodenal papilla for 0.3 cm, the catheter was inserted into the pancreatic duct and a 5F (1.67 mm diameter, 7 cm length) plastic stent was placed using a guidewire (Fig. 1B). After the trocar was placed, the gastrocolic ligament and hepatogastric ligament were transected under laparoscopic view. After the transverse colon was separated from the descending colon and transverse duodenum (Fig. 1C), the pancreas was explored using intraoperative ultrasound (Fig. 1D). The tumor was removed using an ultrasonic cutter, and sharp dissection was used where the tumor abutted against the main pancreatic duct (Fig. 1E). A suture ligation was performed for bleeding or suspected pancreatic leakage in the transverse section of the pancreas, and a drainage tube was placed at the pancreatic incision. The procedure took 138 minutes with an intraoperative blood loss of approximately 10 mL. Intraoperative pathology suggested a well-differentiated pancreatic neuroendocrine tumor (Fig. 1F, stage G1).

The patient was given a liquid diet from the first postoperative day and was discharged on the sixth postoperative day. The abdominal drainage tube was removed on the 15th postoperative day. Abdominal CT plain (Fig. 1G) showed that the position of the pancreatic duct stent was good, and the pancreatic duct stent was removed under ERCP guidance 1 month after surgery (Fig. 1H and I). At 10 months postoperatively, the morphology of the pancreas was normal and no localized stenosis or dilatation of the pancreatic duct was detected.

### 2.2. Case 2

Case 2 was a 60-year-old woman who was admitted to the hospital with complaints of tinnitus for 1 week. Enhanced CT of the upper abdomen detected an uncinate occupying pancreatic lesion, and a diagnosis of the neuroendocrine tumor was made. MRI and magnetic resonance cholangiopancreatography showed a pancreatic wedge-shaped mass measuring approximately 1.9 cm, which was tightly adherent to the main pancreatic duct.

The patient underwent the same perioperative management as Case 1. The surgery lasted 150 minutes, with intraoperative blood loss of approximately 20 mL. Intraoperative pathology was suggestive of a well-differentiated pancreatic neuroendocrine tumor (stage G1). The patient was discharged on postoperative day 7, and the abdominal drain was removed on postoperative day 10. Three months postoperatively, the pancreatic duct stent was removed under ERCP guidance. No local stenosis or dilatation of the pancreas was detected during the follow-up.

### 2.3. Case 3

Case 3 was a 21-year-old woman, admitted with complaints of epigastric pain and abdominal distension for 1 day. On physical examination, she was found to have upper abdominal pressure pain. Routine blood tests revealed elevated leukocytes (10.72 × 10^9^/L) containing 92.2% neutrophils. Ultrasound and enhanced CT revealed a 6 cm mass located in the head of the pancreas. MRI showed an enhanced round tumor located near the main pancreatic duct. A diagnosis of infected solid pseudopapillary tumor of the pancreas was made. The patient underwent the same perioperative management as Case 1.

The procedure took about 130 minutes and the estimated blood loss was 30 mL. Postoperative pathology suggested a diagnosis of solid pseudopapillary tumor of the pancreatic head with hemorrhage, necrosis, and cystic changes. The patient was discharged on postoperative day 7 and the abdominal drainage tube was removed on postoperative day 15. The pancreatic duct stent was removed 3 months postoperatively, and no local stenosis or dilatation of the pancreatic duct was detected during follow-up.

## 3. Discussion

In the present cases, pancreatic tumor resection in close adhesion to the main pancreatic duct guided by 3D laparoscopy and intraoperative ultrasound were performed, and no local stenosis or dilatation of the pancreatic ducts was found during follow-up. These results indicated that 3D laparoscopy combined with intraoperative ultrasound technology might be a promising precision surgical method for the treatment of borderline pancreatic tumors.

Traditional surgical interventions, such as pancreaticoduodenectomy and pancreatectomy, are prone to postoperative complications such as new-onset diabetes and defective pancreatic exocrine function. With the popularity of “precision surgery,” tumor resection guided by 3D laparoscopy and intraoperative ultrasound technology has become one of the preferred treatments for borderline pancreatic tumors.^[3–5]^

Compared with the traditional 2-dimensional laparoscopy, the 3D laparoscopic technique provides 3D visual information and higher magnification, which improves the surgical field of view and thus facilitates the determination of distances and relationships between different organs and tissues and helps to improve surgical precision. Endoscopic pancreatic duct stenting and 3D laparoscopic pancreatic tumor resection have realized the concept of “precision surgery.” The procedure preserves as much pancreatic tissue as possible and avoids complex gastrointestinal reconstruction. This can shorten the surgery time, reduce intraoperative blood loss and accelerate postoperative recovery.

Pancreatic tumors are relatively deep in the abdominal cavity and the relationship between the pancreatic duct and bile duct is close. If the bile duct is stented before surgery, bile duct injury can be prevented. Intraoperative ultrasound combined with pancreatic duct stenting can quickly and accurately clarify the relationship between the tumor and the main pancreatic duct, bile duct, and blood vessels, and assist in removing the tumor intraoperatively and ensuring that no residual tumor cells remain after resection. When the tumor is far from the main pancreatic duct and bile duct, an ultrasonic cutter can be used to separate the pancreatic parenchyma and reduce the amount of bleeding. When the tumor is close to the main pancreatic duct, sharp dissection can avoid thermal damage to the main pancreatic duct and bile duct. In addition, endoscopic ultrasound is the most sensitive method to determine benign and malignant pancreatic tumors and to take preoperative biopsies.^[6]^ The most significant risk after pancreatic tumor resection is pancreatic leakage.^[7]^ A study showed that a distance of <2 mm between the tumor and the main pancreatic duct was an independent risk factor for postoperative pancreatic leakage.^[8]^ Although 3 patients in this study had tumors close to the main pancreatic duct, we used ultrasound with preoperative pancreatic duct stenting and 3D laparoscopy to accurately localize the main pancreatic duct and pancreatic tumor intraoperatively. Pancreatic duct stenting allows postoperative drainage of the pancreas, avoiding pancreatic duct hypertension and pancreatitis, and it further reduces pancreatic fluid leakage from small branches of the pancreatic duct. According to the international definition and grading criteria for pancreatic leakage, the 3 patients in this study showed only biochemical leakage because they did not have bleeding, pancreatitis, or abdominal infection, further indicating that the use of ultrasound intraoperatively with preoperative pancreatic duct stenting and 3D laparoscopy can reduce postoperative pancreatic leakage.

There is no consensus regarding the timing of pancreatic duct insertion or resection.^[9]^ According to our experience, tumor resection can be performed 1 day after stent placement if there is no post-insertion pancreatitis, bleeding, or perforation.^[10]^ The decision to remove the stent must be patient-specific. Premature stent removal increases the risk of pancreatic leakage but may cause stent-related complications if left in place for too long. As in the first case reported, we recommend removing the stent as soon as possible after any pancreatic leakage has healed, if the pancreatic duct is not damaged. If the pancreatic duct has been damaged, we recommend keeping the stent in place for 3 to 6 months to avoid pancreatic duct stenosis.

In conclusion, this study reported 3 cases with pancreatic tumors and underwent endoscopic pancreatic duct stenting combined with 3D laparoscopic pancreatic tumor resection, no local stenosis or dilatation of the pancreatic ducts was found during follow-up. Future larger prospective studies are necessary to validate the effectiveness and safety of this procedure.

## Acknowledgments

Written informed consent was obtained from the patients for this study and the authors would like to give sincere appreciation to the patients for their approval of the manuscript.

## Author contributions

**Conceptualization:** Bo Wu, Shi'an Yu.

**Formal analysis:** Xuemin Li, Xiaokang Wu.

**Writing – original draft:** Bo Wu, Yang Bai.

**Writing – review & editing:** Bo Wu, Yang Bai, Shi'an Yu, Xuemin Li, Xiaokang Wu.
